# Prognostic value of cervical nodal tumor volume in nasopharyngeal carcinoma: Analysis of 1230 patients with positive cervical nodal metastasis

**DOI:** 10.1371/journal.pone.0176995

**Published:** 2017-05-10

**Authors:** Fo-Ping Chen, Guan-Qun Zhou, Zhen-Yu Qi, Li Lin, Jiang Hu, Xiao-Ju Wang, Ying Sun

**Affiliations:** State Key Laboratory of Oncology in Southern China, Collaborative Innovation Center of Cancer Medicine, Department of Radiation Oncology, Cancer Center, Sun Yat-sen University, Guangzhou, People’s Republic of China; University of North Carolina at Chapel Hill, UNITED STATES

## Abstract

**Purpose:**

To investigate the variability and prognostic value of nodal tumor volume (NTV) in nasopharyngeal carcinoma (NPC).

**Methods and materials:**

Data on 1230 patients with newly diagnosed stage T1-4N1-3M0 NPC treated with definitive radiation therapy with or without chemotherapy at a single cancer center were reviewed. NTV was determined from dose volume histogram (DVH) data. X-tile analysis was applied to identify the optimal cut-off points for the NTV with respect to regional recurrence-free survival (RRFS). Correlations between the TNM classification system, NTV and RRFS were assessed using a Cox regression model. Cross-validation based on receiver operating characteristic (ROC) curve analysis was applied to compare the prognostic predictive validity of NTV and N categories.

**Results:**

Within a median follow-up of 49.9 (range, 1.27–76.40) months, 61/1230 (5%) patients developed regional recurrence and 154 (12.5%) developed distant metastasis. NTV values of 7.2 cc and 35.7 cc were identified as the optimal cut-off points. Patients with larger NTV had poorer prognosis. Compared with the N category, NTV was better at determining RRFS for patients with NPC. Hazard ratios increased with NTV, ranging from 1.86 (95% confidence interval [95% CI], 0.92–3.78) for NTV between 7.2 cc to 35.7 cc, and 3.67 (95% CI, 1.58–8.50) for NTV > 35.7 cc. With both NTV and N category in the same Cox regression model, only NTV remained statistically significant in the RRFS of NPC. The validation results with ROC curves also revealed that, NTV was superior to N category for predicting RRFS with significantly larger area under the ROC curve.

**Conclusions:**

NTV offers important prognostic value for treatment outcomes in NPC, especially regional control. Volumetric analysis of nodal involvement may assist selection of patients with poor prognosis.

## Introduction

Nasopharyngeal carcinoma (NPC) is endemic in southern China, northern Africa and Alaska, yet relatively rare in most other regions of the world [1]. Due to its non-specific symptoms and concealed location, most patients are diagnosed with loco-regionally advanced NPC and up to 85% of patients have regional lymph node metastasis at diagnosis [2–3]. Accurate evaluation of cervical nodal metastasis is critical in order to select the appropriate therapeutic schedule and predict prognosis in patients with squamous cell carcinomas of the head and neck, including NPC [3].

Tumor bulk is well-recognized as a major prognostic factor in cancer, as a larger tumor bulk indicates a heavier tumor burden [4–6]. Tumor bulk is associated with several adverse factors for treatment outcomes, such as inherent tumor resistance to radiation treatment, the presence and extent of tumor hypoxia, and metastatic potential [7–8]. The prognostic significance of nodal tumor volume (NTV) has been recognized and adopted in the TNM staging system for NPC by employing a crude measurement of nodal diameter as well as assessment of nodal level and laterality [9]. However, such methods of assessing NTV may vary considerably between different clinicians, as consideration of the “longest minor axis” is extremely subjective.

To date, the prognostic value of the NTV in patients with NPC remains poorly characterized. Therefore, we undertook a large-scale study with the aim of delineating the relationship between the NTV and the treatment outcomes of patients with NPC. This work may allow us to refine the staging systems and treatment strategies for NPC.

## Materials and methods

### Patient selection

A prospectively-maintained database on 1230 consecutive patients with newly biopsy-proven, newly diagnosed stage T1-4N1-3M0 NPC, treated with definitive IMRT at our center between October 2009 and February 2012 was retrospectively analyzed. Electronic medical records and imaging data of each patient were collected. All patients were prospectively followed-up according to a defined routinely out-patient schedule. Telephone follow-up was performed when necessary. All patients underwent pretreatment evaluations including a complete physical examination, hematology and biochemistry profiling, neck and nasopharyngeal MRI, chest radiography or computed tomography (CT), abdominal ultrasonography, whole body bone scan (ECT) and dental assessment. All patients were restaged according to the 7th edition of American Joint Commission on Cancer (AJCC) / International Union Against Cancer (UICC) staging system [9]. The detailed clinicopathological characteristics and treatment factors of the patients are listed in Table 1. This retrospective study was approved by the institutional Ethical Board of the Sun Yat-sen University Cancer Center, and all methods were carried out in accordance with the guidelines of the World Medical Association (WMA) Declaration of Helsinki.

**Table 1:** Patient characteristics and treatment factors.

Characteristic	Patients (%)
*Patient and tumor characteristics*	
Age (years)	
Median	44
Range	14–78
Sex	
Male	916 (74.5)
Female	314 (25.5)
WHO histologic type	
Differentiated	67 (5.4)
Undifferentiated	1163 (94.6)
T category	
T1	174 (14.1)
T2	177 (14.4)
T3	639 (52.0)
T4	240 (19.5)
N category	
N1	841 (68.4)
N2	248 (20.2)
N3	141 (11.5)
Clinical stage	
II	243 (19.8)
III	630 (51.2)
IV	357 (29.0)
*Treatment factors*	
IMRT prescribed dose	
GTVp (Gy/Fr)	66-75/28-35
GTVnd (Gy/Fr)	60-72/28-35
Chemotherapy, *n* (%)	
None	96 (7.8)
CCRT + /− ACT	463 (37.7)
CCRT + NCT	522 (42.4)
NCT + /− ACT	149 (12.1)

Abbreviation: GTVp: primary nasopharyngeal gross tumor volume; GTVnd: tumor volume for involved cervical lymph nodes; Gy/Fr: Gray/Fraction; CCRT: concurrent chemoradiotherapy; ACT: adjuvant chemotherapy; NCT: neoadjuvant chemotherapy.

### Radiotherapy

All patients received intensity-modulated radiation therapy (IMRT) as the primary treatment modality. The patients were immobilized using a thermoplastic head and shoulder mask in a supine treatment position. Contrast-enhanced CT scans depicting the area from the superior border of the frontal sinus to 2 cm below the sterno-clavicular joint were obtained at a 3 mm-slice thickness. CT images were transferred to the Monaco treatment planning system (version 3.02; Elekta AB, Stockholm, Sweden).

Target volumes and normal tissues were delineated on each slice on the treatment-planning system in agreement with the International Commission on Radiation Units and Measurements Reports 62 and 83 [10–11]. The gross tumor volume (GTV) included the primary nasopharyngeal tumor (GTVp) and involved lymph nodes (GTVnd). The GTV was delineated according to clinical, endoscopic and radiological examination findings. Two clinical target volumes (CTV1 and CTV2) were delineated based on the GTV. CTV1 was defined as the high-risk region that encompassed the GTVp plus a 5–10 mm margin, including the entire nasopharyngeal mucosa and a 5 mm submucosal region, and CTV2 as a low-risk region that contained the CTV1 plus a 5–10 mm margin (3–5 mm margin posteriorly) to encompass sites of microscopic extension and the lymphatic regions. The low-risk microscopic extension covered in the CTV2 included the parapharyngeal spaces, posterior third of the nasal cavities and maxillary sinuses, pterygoid processes, pterygoid fossae, base of skull, lower half of sphenoid sinus, anterior half of clivus and petrous apex. The lymphatic regions including the retropharyngeal lymph nodal regions and bilateral levels II, III and Va were routinely delineated in all patients, whereas the ipsilateral levels IV, Vb and supraclavicular fossae were also covered if positive lymph nodes were located below level II. Planning target volumes (PTVs) for all GTVs and CTVs were constructed automatically by expanding the corresponding GTVs and CTVs by 3–5 mm according to immobilization and localization uncertainties.

The prescribed doses to the PTVp, PTVnd, PTV1 and PTV2 were 66–72 Gy, 64–70 Gy, 60–63 Gy and 54–56 Gy, respectively, in 28–33 fractions, with the dose to the organs at risk minimized within the recommended constraints without affecting target coverage [12]. All patients were treated following a routine schedule with one fraction daily over 5 days per week.

### Chemotherapy

Institutional guidelines recommended radiotherapy alone for stage I NPC, concurrent chemoradiotherapy (CCRT) for stage II, and CCRT + /− neoadjuvant/adjuvant chemotherapy (ACT) for stage III to IVA-B. Of the 1230 patients, 96 (7.8%) were treated with IMRT alone; 985 (80.1%) received concurrent chemotherapy, among whom 522 (42.4%) received neoadjuvant chemotherapy, and 22 (1.8%) received adjuvant chemotherapy; 149 (12.1%) were treated with neoadjuvant chemotherapy + /− adjuvant chemotherapy. Neoadjuvant or adjuvant chemotherapy consisted of cisplatin with 5-fluorouracil, platinum with taxoids or platinum with both 5-fluorouracil and taxoids, administered at intervals of 3 weeks for a total of two to four cycles. Concurrent chemotherapy was the single drug platinum administered every three weeks or weekly, beginning on first day of IMRT.

### Follow-up

After completion of IMRT, all patients were followed-up according to a routine schedule with at least every 3 months in the first 2 years and every 6 months thereafter. During every follow-up visit, disease status and treatment toxicities were assessed via routine follow-up care, including complete head and neck examination, hematology and biochemistry profiles, chest radiography, ECT and abdominal sonography. Follow-up nasopharyngeal and/or neck MRI was performed every 6–12 months, especially if residual tumor or recurrence were suspected or new neurological symptoms occurred.

Primary end-point was regional recurrence-free survival (RRFS), calculated from start of treatment until regional recurrence (tumor residue or recurrence at least 3 months after initial IMRT) or last known date free of regional recurrence. Regional recurrence was diagnosed via fine-needle aspiration or MRI or CT of neck and clinical examination of neck.

### Statistical analyses

The dose volume histogram (DVH) data for all patients were reviewed. NTV was identified as the volume of the GTVnd obtained from DVH data. Patients were stratified into subsets based on the NTV using X-tile analysis. X-tile analysis is a graphical method that provides a single, global assessment of every possible method of dividing a population into low-, medium-, and high-level subpopulations [13]. The X-tile software also divides a single cohort into training and validation subsets at a ratio of 1:1 for *P*-value estimation. The optimal cut-off points for the NTV with respect to RRFS were determined by locating the brightest pixel on the X-tile plot of the training cohort. Statistical significance was determined by applying the defined cut-off points to the validation cohort. In addition, the software provides standard Monte Carlo simulations (e.g., cross-validation) for assessing statistical significance by producing corrected *P*-values.

The patients were divided into two groups by age: young age (≤ 44 years) group and old age (> 44 years) group. Receiver operating characteristic (ROC) curve analysis was applied to determine optimal cut-off points, which were defined by maximizing the conditional Youden score, for Epstein–Barr virus (EBV) Deoxyribose Nucleic Acid (DNA) load with respect to regional recurrence. The Kruskal-Wallis test was used to test for differences in NTV among the various N stages. A Cox proportional hazards regression model was applied to assess the correlations between TNM staging system, NTV and the prognosis of patients with NPC while controlling for age, gender, cervical nodal necrosis (CNN), extracapsular spread (ECS) and chemotherapy.

ROC analysis also was applied to compare the predictive validity of NTV and N category associated with RRFS from NPC. The procedure for ROC curve analysis was as follows: First, the total cohorts were divided randomly into two groups, with two-thirds test cohort for training regression coefficients and one-third validated cohort to predict events for each individual. The numbers of predicted events were then compared with the numbers of observed events by goodness of fit based on Pearson chi-square or deviance tests. Sensitivity and false-positive rates were calculated by comparing observed and predicted values using validated cohort, and ROC curves were constructed with sensitivity and false-positive rates as the x-axis and the y-axis, respectively. The area under ROC curve (AUC) was used to assess the predicted validity of NTV and N category based on the method of Hanley and McNeil [14]. Sensitivity, specificity, and AUC calculations for this study were also adjusted for age, gender, nodal radiation dose and chemotherapy.

X-tile plots were constructed using X-tile 3.6.1 (Yale School of Medicine, City of New Haven, CT, USA) [13]. Overall survival (OS), disease-free survival (DFS), RRFS and distant metastasis-free survival (DMFS) were calculated using the Kaplan-Meier method and the differences were compared using the log-rank test. Kruskal-Wallis test was used to compared the difference of NTV among N categories. Univariate and multivariate analyses were performed using SPSS 22.0 (SPSS, Chicago, IL, USA). ROC curve analysis was performed using MedCalc 16.4.3 (MedCalc Software, Ostend, Belgium). All tests were two-sided; *P*-values < 0.05 were considered statistically significant.

## Results

### Patient outcomes and nodal tumor volume analysis

**Fig 1:**
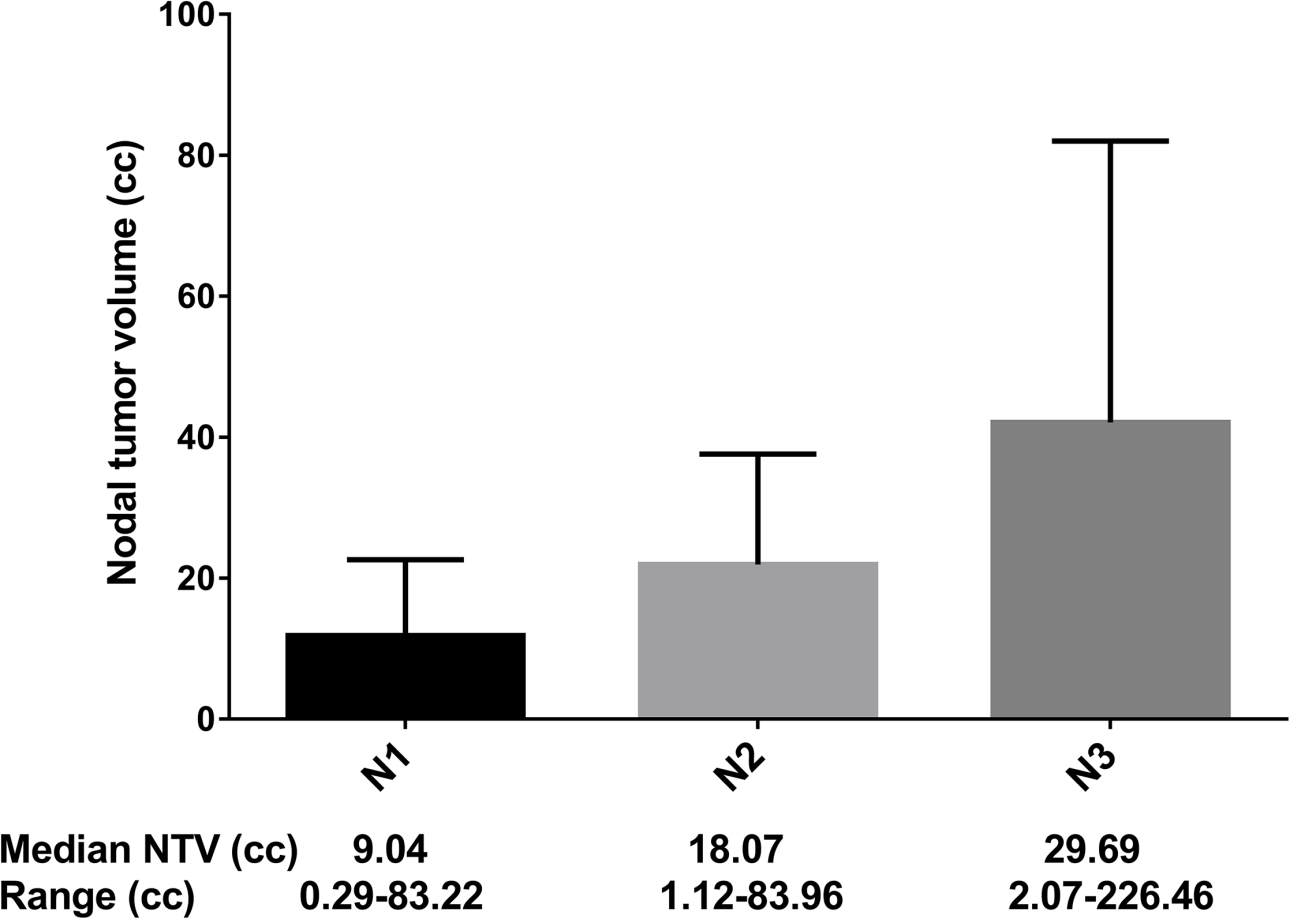
Correlation between nodal tumor volume and N stage of nasopharyngeal carcinoma (NPC).

**Fig 2:**
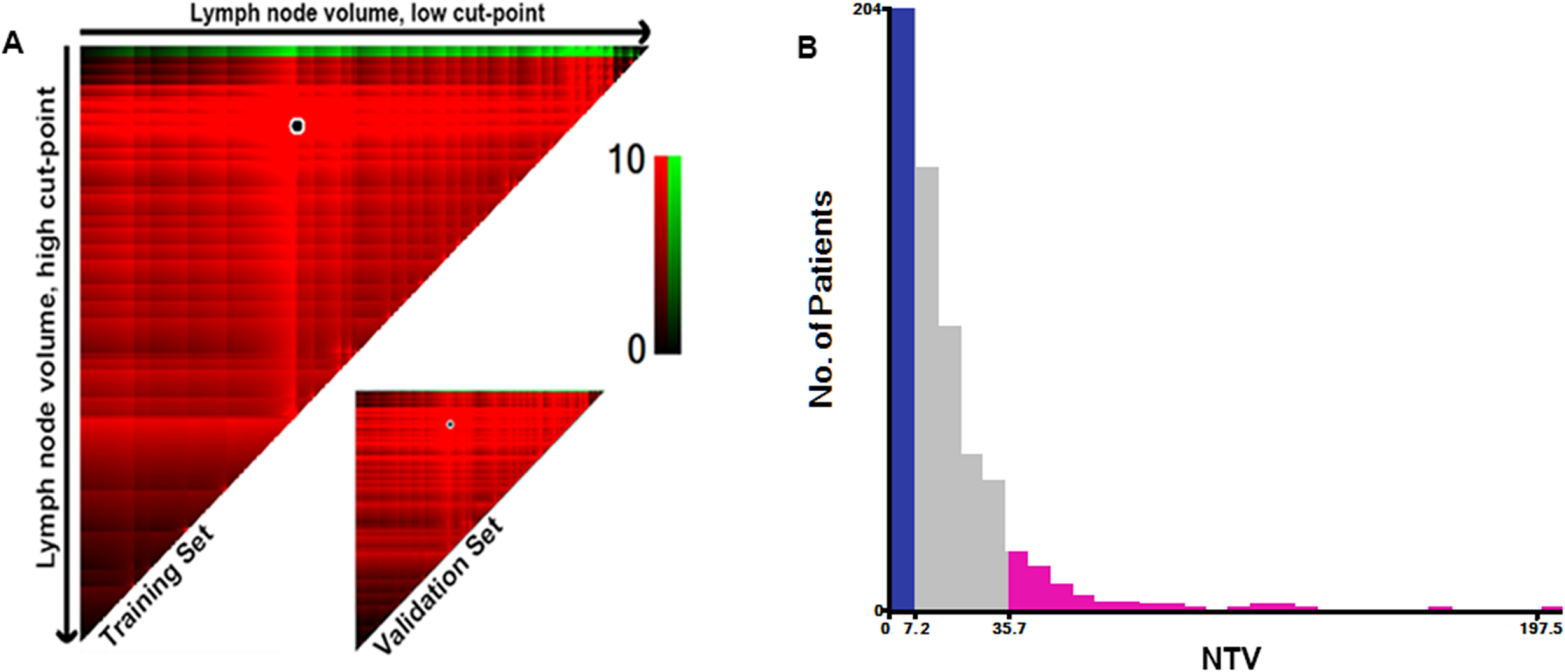
X-tile analysis of 1230 patients with newly diagnosed stage T1-4N1-3M0 nasopharyngeal carcinoma (NPC) reveals a continuous distribution based on nodal tumor volume. A shows nodal tumor volume divided at the optimal cut-points (7.2 cc and 35.7 cc, P = 0.03), as defined by the most significant (brightest pixel) on the plot.

After a median follow-up of 49.9 months (range, 1.27–76.40 months), 61/1230 (5%) patients developed regional recurrence and 154/1230 (12.5%) developed distant metastasis. The median NTV of the whole series was 11.85 cc (range, 0.29–226.46 cc). Fig 1 shows the NTV distribution and for each N category. Although the variation within the same N category was wide, the median NTV increased orderly with advancing N category: N1, 9.04 cc; N2, 18.07 cc; and N3, 29.69 cc (Fig 1; *p* < 0.0001). NTV was found to be associated with regional recurrence in the X-tile plot (Fig 2A). The histogram demonstrated a diffuse continuous distribution with no discernable subpopulations (Fig 2B). X-tile analysis identified 2 optimal NTV cut-points, 7.2 cc and 35.7 cc, with respect to RRFS in the training set. Statistical significance was achieved with a *P*-value of 0.03 when the cut-off points were applied to the validation set.

### Correlation between NTV, N category and survival

**Fig 3:**
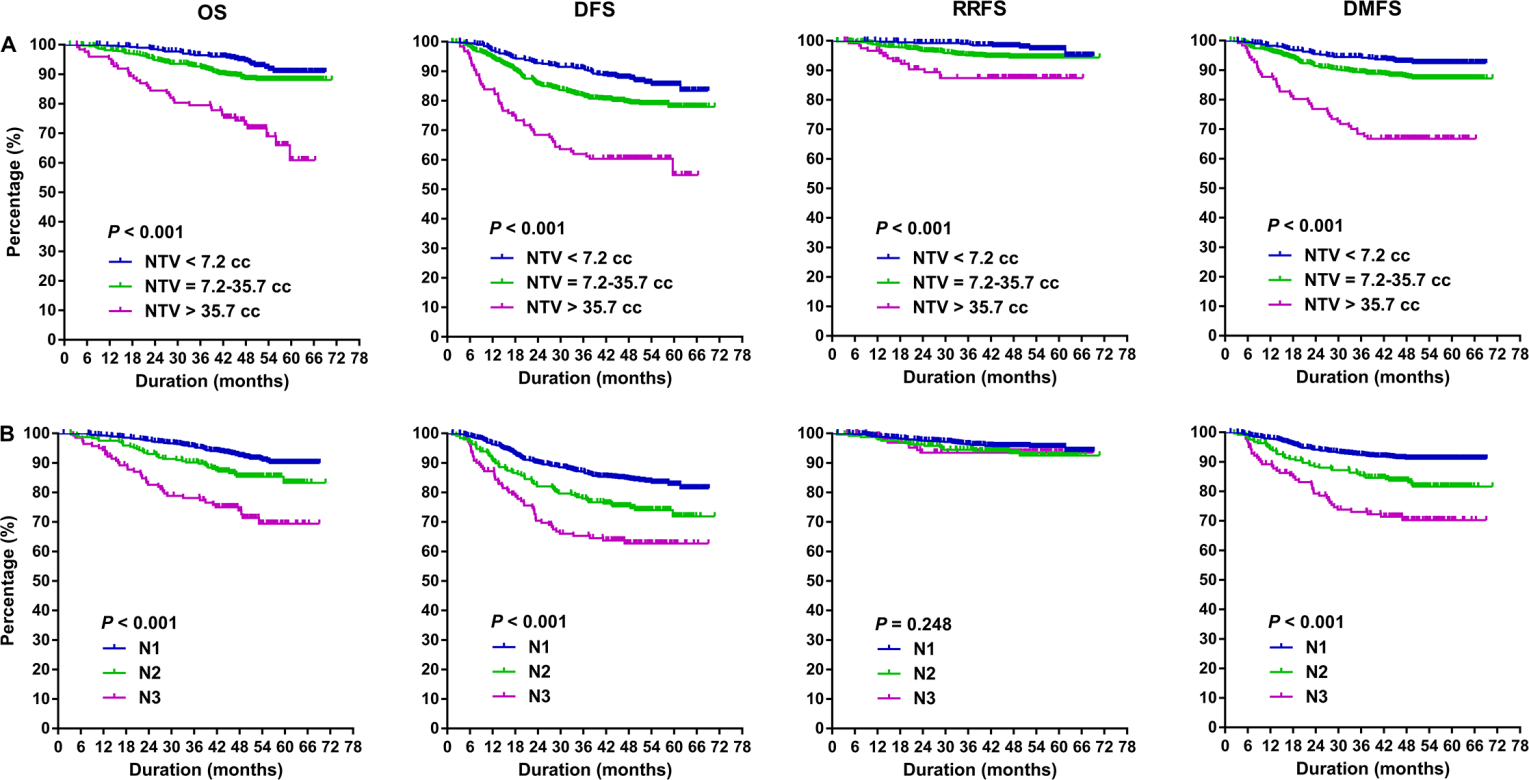
Probability of overall survival (OS), disease-free survival (DFS), regional recurrence-free survival (RRFS) and distant metastasis-free survival (DMFS) according to nodal tumor volume (NTV) and N category in newly diagnosed stage T1-4N1-3M0 nasopharyngeal carcinoma (NPC).

The cumulative survival rates by NTV and N category were presented in Fig 3, which showed that patients with advanced N stage and larger NTV had poorer OS, DFS and DMFS(*P* < 0.001). However, the survival curves separated clearly among NTV subpopulations (*P* < 0.001), while the differences of RRFS among different N categories failed to achieve (*P* = 0.248). Five-year RRFS of low-, medium-, and high-level subpopulations were 94.4%, 94.4% and 87.3% respectively, compared with 93.8%, 92.5% and 93.5% of N1 to N3 respectively.

### Prognostic risk factors analyses

Univariate analysis revealed that NTV, T category, N category, clinical stage, old age (> 44 years); EBV DNA > 2635 copies/mL, differentiated tumors, CNN and ECS were significantly associated with OS and DFS (Table 2). EBV DNA > 2635 copies/mL, NTV, CNN and ECS were identified to be associated with RRFS, and NTV, T category, N category, clinical stage, EBV DNA > 2635 copies/mL, CNN and ECS were associated with DMFS.

**Table 2:** Univariate analysis of variables associated with various clinical endpoints.

Characteristic	No. of Patients^*^	5-Year OS	*P*†	5-Year DFS	*P*†	5-Year RRFS	*P*†	5-Year DMFS	*P*†
Sex			0.180		0.743		0.200		0.449
Male	916 (74.5)	85.4		76.9		93.5		86.5	
Female	314 (25.5)	88.6		78.3		93.3		87.5	
Age			<0.001		0.009		0.901		0.133
≤ 44 years	622 (50.6)	90.8		82.1		94.3		88.1	
> 44 years	608 (49.4)	81.4		71.8		92.2		85.3	
Smoking			0.120		0.423		0.595		0.863
Yes	467 (38)	84.1		75.0		92.5		86.8	
None	763 (62)	87.6		78.8		94.2		86.7	
EBV-DNA			<0.001		<0.001		0.002		<0.001
≤ 2635 copies/mL	581 (47.2)	90.8		82.8		94.4		92.3	
> 2635 copies/mL	649 (52.8)	81.2		71.1		92.6		80.6	
Histology			0.001		0.013		0.117		0.145
Differentiated	67 (5.4)	70.4		68.7		90.4		81.6	
Undifferentiated	1163(94.6)	87.3		77.7		93.6		97.8	
T category			<0.001		0.002		-		0.015
T1	174 (14.1)	94.4		74.5				89.9	
T2	177 (14.4)	88.3		82.8				88.4	
T3	639 (52)	87.8		80.2				87.9	
T4	240 (19.5)	74.9		68.2				80.4	
N category			<0.001		<0.001		0.248		<0.001
N1	841 (68.4)	89.6		81.1		93.8		91.0	
N2	248 (20.2)	84.0		72.2		92.5		81.2	
N3	141 (11.5)	69.5		62.3		93.5		70.4	
Clinical stage			<0.001		<0.001		-		<0.001
II	243 (19.8)	96.0		81.8				93.3	
III	630 (51.2)	89.4		81.6				89.4	
IV	357 (29)	74.0		66.6				77.5	
NTV			<0.001		<0.001		<0.001		<0.001
≤ 7.14 cc	404 (32.8)	90.2		82.5		94.4		92.3	
7.15–35.71 cc	701 (57)	88.2		78.2		94.4		87.1	
> 35.71 cc	125 (10.2)	62.3		56.5		87.3		66.8	
CNN			0.001		<0.001		<0.001		<0.001
Yes	385 (31.3)	80.4		69.1		90.4		81.6	
None	845 (68.7)	88.9		81.0		94.9		89.1	
ECS			<0.001		<0.001		0.005		<0.001
Yes	377 (39.7)	78.6		71.0		91.4		80.2	
None	853 (69.3)	89.6		79.9		94.2		89.6	
Chemotherapy, *n* (%)			0.812		0.963		0.865		0.914
None	96 (7.8)	90.5		72.8		95.0		86.4	
CCRT + /− ACT	463 (37.7)	85.2		77.5		95.2		81.6	
CCRT + NCT	522 (42.4)	86.5		78.5		94.3		81.8	
NCT + /− ACT	149 (12.1)	85.9		77.6		94.1		92.9	

Abbreviation: EBV: Epstein–Barr virus; NTV: nodal tumor volume; CNN: cervical nodal necrosis; ECS: extracapsular spread; DFS: disease-free survival; DMFS: distant metastasis–free survival; OS: overall survival; RRFS: regional recurrence-free survival; CCRT: concurrent chemoradiotherapy; ACT: adjuvant chemotherapy; NCT: neoadjuvant chemotherapy.

* Data in parentheses are percentages.

† P-values were calculated using the log-rank test.

Multivariate analysis revealed that NTV, T category, N category, age (> 44 years), EBV DNA (> 2635 copies/mL) and differentiated tumors were significant independent negative prognostic factors for OS, and these factors—in addition to CNN—also achieved prognostic significance for DFS (Table 3). EBV DNA > 2635 copies/mL, NTV and CNN were independent adverse prognostic factors for RRFS, and EBV DNA > 2635 copies/mL, T category, N category and NTV were established as independent adverse prognostic factors for DMFS. However, NTV remained an independent prognostic factor for RRFS, while N category failed to achieve prognostic significance. This indicated that NTV might be a better predictor of RRFS for patients with NPC. Chemotherapy status (RT alone vs. CCRT + /− ACT vs. CCRT + NCT vs. NCT + /− ACT) did not significantly affect survivals in the entire cohort.

**Table 3:** Multivariate analysis of variables associated with various clinical endpoints.

Characteristic	5-Year OS		5-Year DFS		5-Year RRFS		5-Year DMFS	
	Hazard ratio	*P*†	Hazard ratio	*P*†	Hazard ratio	*P*†	Hazard ratio	*P*†
Sex		NS		NS		NS		NS
Age (> 44 years)	1.78 (1.26–2.52)	0.001	1.35 (1.05–1.74)	0.019		-		-
Smoking		NS		NS		NS		NS
EBV-DNA (> 2635 copies/mL)	1.53 (1.07–2.21)	0.022	1.52 (1.15–1.99)	0.003	1.72 (1.01–2.95)	0.050	1.98 (1.38–2.83)	<0.001
Histology (undifferentiated)	0.48 (0.29–0.79)	0.004	0.59 (0.37–0.92)	0.021		-		-
T category						-		
T1	Ref.		Ref.				Ref.	
T2	1.39 (0.61–3.16)	0.438	0.83 (0.50–1.38)	0.479			0.94 (0.48–1.80)	0.847
T3	1.87 (0.93–3.76)	0.080	0.97 (0.65–1.44)	0.863			1.02 (0.60–1.73)	0.954
T4	3.88 (1.88–8.01)	<0.001	1.59 (1.03–2.47)	0.038			1.80 (1.01–3.21)	0.045
N category						NS		
N1	Ref.		Ref.				Ref.	
N2	1.27 (0.83–1.95)	0.277	1.24 (0.91–1.71)	0.179			1.51 (1.01–2.26)	0.043
N3	2.24 (1.41–3.56)	0.001	1.68 (1.16–2.43)	0.006			2.16 (1.38–3.36)	0.001
NTV								
≤ 7.14 cc	Ref.		Ref.		Ref.		Ref.	
7.15–35.71 cc	1.72 (1.09–2.69)	0.019	1.50 (1.08–2.07)	0.016	1.86 (0.92–3.78)	0.084	1.51 (0.98–2.34)	0.065
> 35.71 cc	3.41 (1.93–6.04)	<0.001	2.45 (1.57–3.83)	<0.001	3.67 (1.58–8.50)	0.002	3.20 (1.85–5.54)	<0.001
CNN		NS	1.44 (1.11–1.87)	0.007	2.06 (1.22–3.46)	0.007		NS
ECS		NS		NS		NS		NS
Chemotherapy, n (%)		NS		NS		NS		NS
None								
CCRT + /− ACT								
CCRT + NCT								
NCT + /− ACT								

Abbreviation: Ref.: reference value; NS: not significant; EBV: Epstein–Barr virus; NTV: nodal tumor volume; CNN: cervical nodal necrosis; ECS: extracapsular spread; DFS: disease-free survival; DMFS: distant metastasis–free survival; OS: overall survival; RRFS: regional recurrence-free survival; CCRT: concurrent chemoradiotherapy; ACT: adjuvant chemotherapy; NCT: neoadjuvant chemotherapy.

Note: Hazard ratios and P-values were calculated using the Cox proportional hazards model. Numbers in parentheses are 95% confidence intervals.

† P-values were calculated using the log-rank test.

### Prognostication of RRFS using NTV and N category

**Fig 4:**
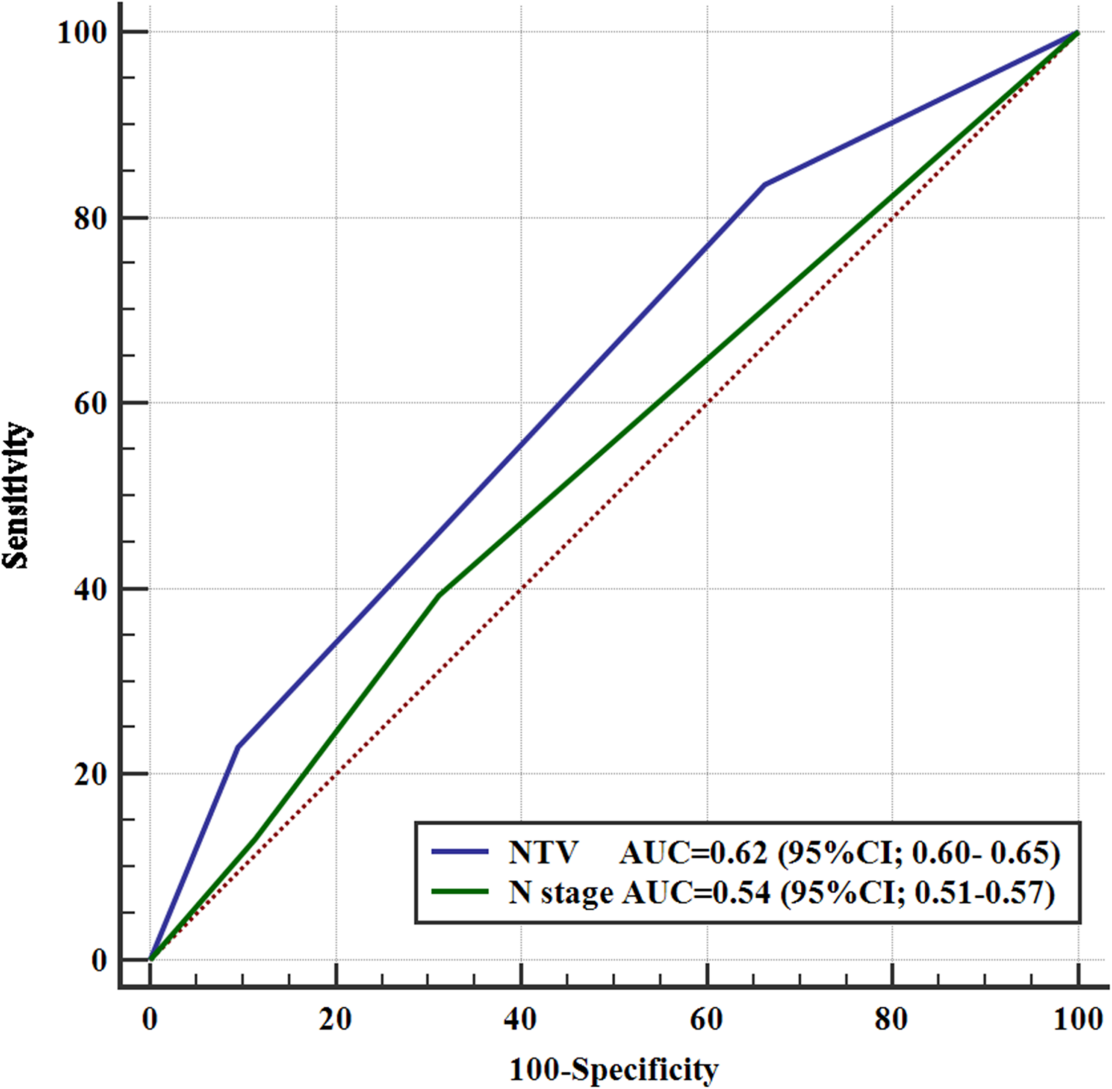
Receiver operating characteristic (ROC) curves of nodal tumor volume (NTV) and N category for predicting regional recurrence-free survival.

To verify whether the predictive validity for NTV was better compared with N category, cross-validation was performed as shown in Fig 4. NTV was superior to N category for predicting RRFS; the area under the ROC curve was 0.62 (95% confidence interval [95% CI], 0.60–0.65) for NTV compared with 0.54 (95% CI, 0.51–0.57) for N category (*P* = 0.016). After adjusting for age, gender, nodal radiation dose and chemotherapy, the AUC was 0.65 (95% CI, 0.62–0.67) for NTV and 0.56 (95% CI, 0.53–0.59) for N category (*P* = 0.024).

## Discussion

NPC has a high propensity for cervical node metastasis [2–3]. Francis et al. [2] conducted a meta-analysis of 13 clinical trials that used MRI for diagnosis and staging, which concluded that the most commonly involved cervical lymph node regions in NPC include the lateral retropharyngeal nodes and level II nodes, with overall probabilities of metastasis of 69.4% and 70.4%, respectively. The pattern of nodal metastases in NPC has been firmly established [15–17], with the common view that cervical node metastases occur in an orderly fashion. The aforementioned first echelon nodal groups reported by Francis et al. [2] are followed by levels III, VA and IV, with gradually reduced probabilities of 44.9%, 26.7% and 11.2%, respectively. Moreover, there is a very low risk of skip nodal metastasis, with a probability of 0.5% [18].

The TNM stage at presentation is the most commonly used prognostic indicator in NPC [19–20]. N category of the AJCC staging system has been proven to be associated with regional control and distant metastasis [20]. Despite such proven value, the N category of NPC staging is defined by the extent of lymph node involvement according to the involved cervical lymph node regions, in addition to the maximum diameter of the lymph nodes, and this method has several limitations. It has been suggested that NTV may have significant prognostic value [4–5].

In this study, we demonstrated that the NTV is significantly associated with OS, DFS, RRFS and DMFS in NPC. Multivariate analysis confirmed that NTV was significantly associated with adverse regional control, whereas N category had no prognostic significance for regional control after adjusting for cofounders. Moreover, ROC curve analysis further verified the superior prognostic ability of the NTV over N category. However, our data suggested that both the NTV and N category were significant independent negative prognostic factors for OS, DFS and DMFS.

The prognostic significance of the NTV for the treatment outcomes of patients with NPC has previously been assessed. In a retrospective analysis of 290 patients, Chua et al. [4] found that a NTV > 30 cc was associated with a significantly higher distant failure rate (5-year distant relapse-free survival: 54%) and lower disease-specific survival (5-year DSS: 40%). Furthermore, they evaluated the prognostic value of the NTV in patients with early-stage NPC treated using radiotherapy alone [5], and found that a NTV > 4 cc was associated with a lower 5-year distant failure-free rate (5-year distant failure-free rate, 72% vs. 90%; P = 0.011) and poorer 5-year DSS (76% vs. 94%; P = 0.0038). However, nodal control was excellent, with no difference between patients with a NTV < 4 cc and > 4 cc (5-year control rate, 97% vs. 100%).

The relationship between NTV and regional control is not simply a matter of the number of tumor cells required to be sterilized. Other important tumor variables, such as tumor hypoxia and radiotherapy resistance, may also affect the treatment outcome. The nodal radiation dose is obviously another important factor that should be considered. However, current researches mainly focused on the dose-volume-control effect of local tumors, which cannot fully directly applied to nodal tumors, as nodal tumor has its own clinical features. Studies zooming in on the dose-volume-control effect of nodal tumors are warranted, which may assist the clinician in optimal dose prescribing.

Certain nodal characteristics such as nodal level, size, and bilaterality, are strong predictors of distant metastases in NPC. The estimation of NTV may also provide predictive value in distant metastases in NPC, as confirmed by our study. In the current study, larger NTV was significantly associated with a higher risk of distant failure, in multivariate analysis.

CNN is commonly seen in NPC, and has been reported to be associated with treatment outcomes in patients with NPC [21]. The reported incidence of CNN ranges from 20% to 42% in NPC [17, 22–23]. Mao et al. [23] reported the incidence of CNN was 26.5% in patients with positive cervical lymph nodes. In a study of 1800 patients, Lan et al. [21] reported a higher incidence of 44.0% of CNN in patients with nodal metastasis, and indicated that CNN is an important independent prognostic factor for OS, DFS, RRFS and DMFS. In this cohort, CNN was observed in 31.3% of the patients with positive nodes, and CNN was found to be an independent prognostic factor for DFS and RRFS. However, CNN failed to achieve significant prognostication of OS and DMFS.

## Conclusion

Measurement of the NTV in NPC may provide important prognostic information for predicting treatment outcomes, especially regional control. Patients with a small NTV achieved excellent regional control, irrespective of N category. Volumetric analysis of the involved nodes in NPC would help clinicians select patients with a poor prognosis.
